# Review of Drug Utilization Studies in Neonatal Units: A Global Perspective

**DOI:** 10.3390/ijerph17165669

**Published:** 2020-08-05

**Authors:** Asma Al-Turkait, Lisa Szatkowski, Imti Choonara, Shalini Ojha

**Affiliations:** 1 Division of Graduate Entry Medicine, School of Medicine, University of Nottingham, Nottingham NG7 2RD, UK; asma.al-turkait@nottingham.ac.uk (A.A.-T.); imti.choonara@nottingham.ac.uk (I.C.); 2 Division of Epidemiology and Public Health, School of Medicine, University of Nottingham, Nottingham NG7 2RD, UK; lisa.szatkowski@nottingham.ac.uk; 3 Neonatal Unit, University Hospitals of Derby and Burton NHS Trust, Derby DE22 3NE, UK

**Keywords:** infants, newborn, care, neonatal intensive, drug use review, antibiotics

## Abstract

Rational prescribing is challenging in neonatology. Drug utilization studies help identify and define the problem. We performed a review of the literature on drug use in neonatal units and describe global variations. We searched databases (EMBASE, CINAHL and Medline) from inception to July 2020, screened studies and extracted relevant data (two reviewers). The search revealed 573 studies of which 84 were included. India (n = 14) and the USA (n = 13) reported the most. Data collection was prospective (n = 56) and retrospective (n = 26), mostly (n = 52) from one center only. Sixty studies described general drug use in 34 to 450,386 infants (median (IQR) 190 (91–767)) over a median (IQR) of 6 (3–18) months. Of the participants, 20–87% were preterm. The mean number of drugs per infant (range 11.1 to 1.7, pooled mean (SD) 4 (2.4)) was high with some reporting very high burden (≥30 drugs per infant in 8 studies). This was not associated with the proportion of preterm infants included. Antibiotics were the most frequently used drug. Drug use patterns were generally uniform with some variation in antibiotic use and more use of phenobarbitone in Asia. This study provides a global perspective on drug utilization in neonates and highlights the need for better quality information to assess rational prescribing.

## 1. Introduction

Prescribing drugs to newborn infants, particularly those born preterm, is a challenge fraught with complexities including lack of evidence-based information about pharmacokinetics and pharmacodynamics of drugs, efficacy and side-effect profiles for some of the most frequently used drugs. Despite this, infants in neonatal care are exposed to many drugs, often off-label, unlicensed and without clear guidance on dosing. The large gaps in knowledge translate into large differences in interpretation of the sparse evidence that is available, leading to wide variations in practice on one hand and the perpetuation of incorrect practices on the other.

Drug-utilization research provides an insight into the pattern of prescribing and is the essential first step towards rational drug use and evidence-based pharmacotherapy [1]. Physicians prescribe drugs not necessarily based on the available evidence but also under influence from psychosocial and circumstantial aspects that impact their decisions [2]. Investigation into the trends and variability of drug use in the neonatal population can provide information that could guide effective strategies to improve prescribing practices and highlight areas for research. Observational studies describing patterns of drug use provide preliminary evidence to support this agenda. Although evidence for medication use in neonates is limited, studies describing drug use are accumulating [3] and emerging evidence suggests wide variations in practices across the globe.

The aim of this study was to conduct an up-to-date comprehensive review of literature to accumulate information from studies describing patterns of drug use in neonatal units and describe variations in the most frequently prescribed drugs across different regions.

## 2. Materials and Methods

Three databases (EMBASE, CINAHL and Medline) were searched from their inception to 20 July 2020 based on the following PICo: population, neonates, infants or newborn (all gestational ages); interest, drug use or drug utilization; and context, neonatal intensive care or neonatal care. A combination of free-text and medical subject headings were applied to each database separately. Various free-text keywords were created and used to complement the Medical Subject Headings (MeSH) terms. For the population search terms, infant* or newborn* or neonate* were used and are defined as infants 0–28 days of age. For the interest/intervention search terms, free-text keywords, a combination of drug use and drug utili?ation was applied. The term utilzation was used to include both utilization or utilisation. The context or the setting free-text keywords used for this review were neonatal intensive care unit* and neonatal unit*. This setting was used as the aim of this review was to provide an updated drug utilization literature review at the level of neonatal intensive care units only. All the previously mentioned free-text keywords were used in addition to the MeSH terms identified in each database separately. The full search strategy is detailed in Appendix A (Table A1). Reference lists were searched to identify any relevant articles. Following the retrieval of the records, titles were reviewed to remove any duplicates before starting to screen the abstracts for inclusion.

All observational studies conducted in neonatal units that reported data on the most frequently prescribed drugs, antibiotics or at least therapeutic groups were included. This includes overall frequently prescribed drugs or pharmacological groups, off-label and or unlicensed drugs or specific pharmacological groups. Studies were excluded if data on drug utilization were not available, if the population included children >28 days old, if maternal rather than infant drug use was reported or if the reports were systematic or other reviews.

All included studies were tabulated (using Microsoft Excel, v15, Microsoft Corporation, Redmond, WA, USA) and data on location of study, inclusion and exclusion criteria, demographics of the included population, number of drugs prescribed per infant, length of stay in neonatal care and the ten most frequently prescribed drugs or pharmacological groups were extracted. Screening and data extraction were completed by two authors (AAT and SO). Quality assessment of the studies was not performed as there is no appropriate tool for the type of studies that are included.

Data extracted, where available, included: country (or countries) where neonatal unit(s) was placed, number of neonatal units included in the study, duration of study, number of infants included, proportion of female participants (calculated as total number of participants—number of males, where only number of males was reported), proportion of preterm infants (defined as born at <37 weeks gestational age), inclusion criteria, exclusion criteria for participants, list of excluded medicinal products, gestational age and birth weight of the participants and drugs received per participant (defined as number of individual drugs received per infant during the entire neonatal care reported). Lists of most frequently used drugs were extracted for all drugs, antibiotics and pharmacological groups, where reported. Data, where available, were sought for indication of use, doses, frequency and duration of administration and adverse effects.

The number of studies that reported a drug as one of its 10 most frequently used is reported as counts. Mean and standard deviation (SD) of number of drugs received per infant were extracted from reports were available. Where the SD was not reported, it was imputed from the available summary statistics (mean, median, interquartile range (IQR), range) and sample size using the process described by Hozo et al. [4]. The correlation between proportion of included preterm infants and number of drugs per infant was calculated using the Pearson’s correlation coefficient test in Stata v17.0.

## 3. Results

**Figure 1:**
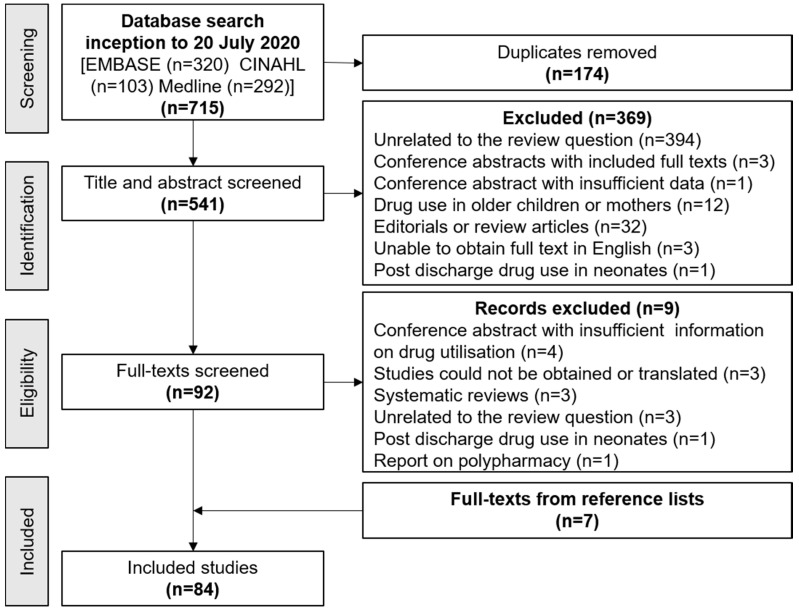
Selection of studies for inclusion.

The search retrieved 715 articles of which 92 were eligible for full-text screening. A description of these studies is given in Table A2, Table A3, Table A4, Table A5, Table A6, Table A7, Table A8, Table A9 and Table A10 in Appendix B. Fifteen further studies were excluded and 7 added from a search of reference lists, such that 84 studies were included in the review. The screening process is illustrated in Figure 1.

### 3.1. Characteristics of Included Studies

Most of the included studies (60/84) evaluated drugs in all drug groups or categories. These 84 included 8 studies that also reported separate analyses of antibiotic use and 20 studies that reported use of off-label medications. In addition, 11 studies reported antibiotic usage only, 6 reported off-label or unlicensed drug use and 7 reported pharmacological groups that were frequently used rather than listing individual drugs. The studies were all observational with 56 prospective and 26 retrospective data collection over a varied time period. Two studies collected both retrospective and prospective data [5,6]. Studies were largely based in a single center (52/84) [5,6,7,8,9,10,11,12,13,14,15,16,17,18,19,20,21,22,23,24,25,26,27,28,29,30,31,32,33,34,35,36,37,38,39,40,41,42,43,44,45,46,47,48,49,50,51,52,53,54,55,56]. Thirty-two studies were based in more than one neonatal unit, ranging from 2 centers (7 studies) [57,58,59,60,61,62,63] to 341 centers (one study) [64].

**Figure 2:**
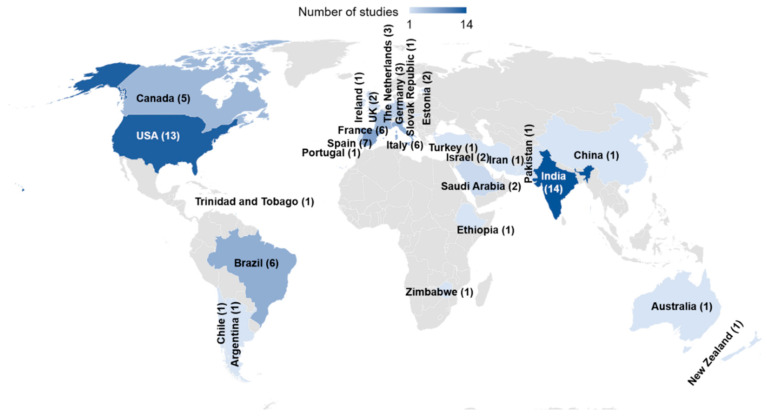
Countries of origin of reports of drug utilization in neonatal units.

Sixty studies, conducted between 1983 and 2020, reported drug use in all therapeutic categories. Most (43 of 60) collected data prospectively while 17 retrieved retrospective data. The studies were conducted in 26 countries (Figure 2) with India and the United States of America (USA) accounting for the largest number of reports, 14 and 13 respectively. There was one study that involved several European countries (21 participated) [65] and one study conducted in Germany and Brazil [66].

The study periods varied from one month [26,55,67,68] to studies spanning over 22 years [33]. The median (interquartile range, IQR) duration of data collection in 79 studies was 6 (3–18) months. Sample size, reported in 77 studies, ranged from 34 [25] to 450,386 [69] infants with a median (IQR) of 190 (91–767) infants. The retrospective studies using large databases with routinely collected data covered the largest span of time and included the largest number of infants, such as Hsieh et al. [69] and Clark et al. [70], who reported data from an administrative electronic database managed by the Pediatrix Medical Group in the USA.

Thirty-four of 60 studies reported the proportion of preterm infants (born at <37 weeks gestational age) among their cohort (range 20% [24] to 87% [71]) in addition to the two studies (34), (31) that included preterm infants only. In addition, one study Puia-Dumitrescu 2020 [72] reported drugs received by infants born at 22–24 weeks gestational age only.

Participants were infants admitted to neonatal units who received at least one drug during their stay. Several studies excluded certain drugs and infusions such as vitamin K, intravenous fluids, parenteral nutrition and fluids used to maintain patency of vascular access. The details of inclusion and exclusion for each included study is given in the tables in Appendix B.

### 3.2. Number of Drugs Per Infant

**Figure 3:**
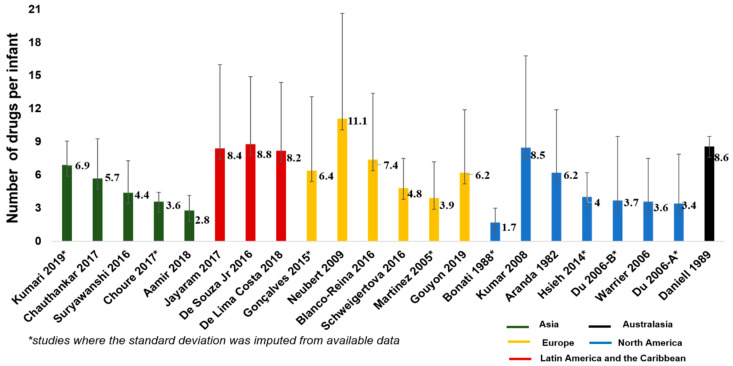
Number of unique drugs per infant reported in drug utilization studies in neonatal units.

**Figure 4:**
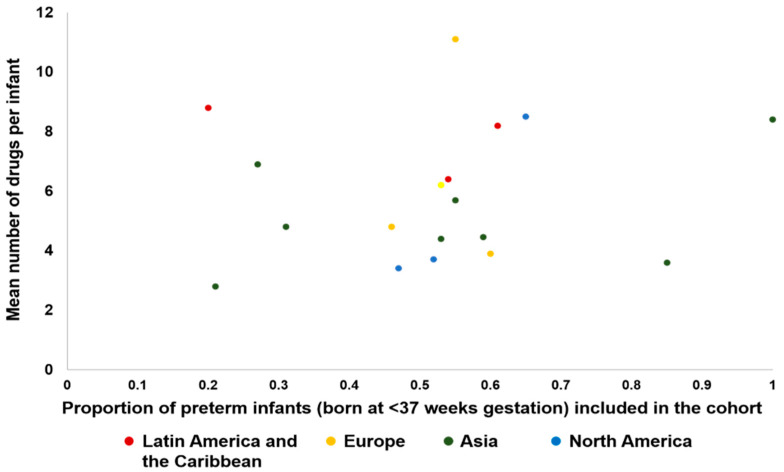
Number of drugs per infant and proportion of preterm infants included in the study.

The mean and standard deviation (SD) of the number of drugs per infants received during neonatal care was reported in 14 studies [8,13,18,22,23,24,34,38,43,51,53,63,73,74] and sufficient information was available to impute the SD value in 7 other studies [19,28,32,39,42,69,75] (Figure 3). The pooled mean (SD) of the number of drugs received per infant, calculated from data reported in 29 studies, was 4 (2.4) drugs. There was no correlation (Pearson’s r = 0.14; *p* value = 0.60) between the number of drugs per infant and the proportion of premature infants included in the studies (Figure 4). Several studies (27 studies) [8,11,13,19,20,22,23,26,28,32,34,37,38,39,42,43,44,45,50,59,63,67,68,69,71,73,75] reported the maximum number of drugs received by at least one infant: Kumar et al. [38] reported the highest drug burden with at least one infant receiving 62 individual drugs, while 8 other studies [13,20,22,23,28,32,43,50] reported that the maximum number of drug per infant was ≥30 in their population.

### 3.3. Most Frequently Prescribed Pharmacological Groups

Thirty out of the 60 included studies reported the most frequently prescribed pharmacological groups, using different methods in their classification. Most used the WHO-Anatomical Therapeutic Chemical (ATC) classification system (19 of 30 studies) [7,13,23,24,25,30,32,34,42,43,50,51,59,63,71,73,76]. Four studies listed the pharmacological class of the drugs [10,19,39,47] while Kumar et al. (2008) [26] classified the pharmacological groups based on the most frequent indication and the physiological effects of the drug (38). The remaining six studies did not state their classification method [14,21,33,61,75,77].

Among the studies that used the WHO-ATC system, anti-infectives for systemic use were the most frequently prescribed pharmacological group in the majority (14 studies) [13,23,24,25,30,34,42,43,50,51,59,71,73,76]. This was followed by agents for the alimentary tract and metabolism (4 studies) [7,63,65,74] and agents for the central nervous system (1 study) (32). Among the four studies that listed the pharmacological groups according to their pharmacological class, three studies reported that antimicrobials were the most frequently prescribed group [19,39,47] and one study by Ashwin et al. (2018) identified that penicillins were the most frequently prescribed [10]. Kumar et al. (2008) reported that the gastrointestinal agents were the most frequently prescribed pharmacological group [38].

### 3.4. Most Frequently Prescribed Drugs

**Figure 5:**
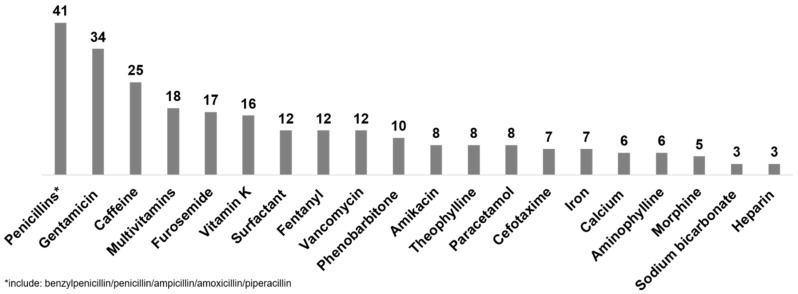
Drugs reported as one of 10 most frequently prescribed in 44 neonatal drug utilization studies. Bars represent the number of studies that reported each drug as one of its 10 most frequently prescribed.

Forty-eight studies reported the most frequently prescribed drugs. Figure 5 shows the drugs and the number of studies that reported it among its list of most frequently prescribed drugs and Table 1 gives a summary of the data by geographic region.

Every study had one or more antibiotic in this list with penicillins (41 studies) and gentamicin (34 studies) reported most frequently. Six studies did not have either penicillin or gentamicin in this list. Of these, two reported antibiotics (without specifying which antibiotics were included) [17,19] and the other four [28,35,48,55] had cefotaxime, ceftriaxone, vancomycin, tobramycin, amikacin. cefoperazone-sulbactam and piperacillin-tazobactam amongst their most frequently prescribed drugs.

Most studies did not report the indications of use, dose, frequency or duration of use or adverse effects of the frequently used drugs.

An antibiotic was the most frequently prescribed drug in most studies. Twenty-one studies reported a drug from another therapeutic class as its most frequently used. These were calcium gluconate (2 studies [7,8]), multivitamins (3 studies [44,65,75]), vitamin K (7 studies [14,22,43,45,55,62,76]), caffeine (2 studies [35,71]), chlorhexidine powder (1 study [37]), theophylline (1 study [42]), epinephrine (1 study [78]), parenteral nutrition (1 study [60]), cholecalciferol (1 study [63]), fentanyl (1 study [32]) and vitamin D (1 study [74]). Of the two studies that reported caffeine as the first most frequently prescribed drug, 86.8% of included infants in Cuzzolin et al. (2016) were preterm [71] while Jong et al. (2001) did not report the preterm proportion in their cohort [35].

### 3.5. Most Frequently Prescribed Antibiotics

**Figure 6:**
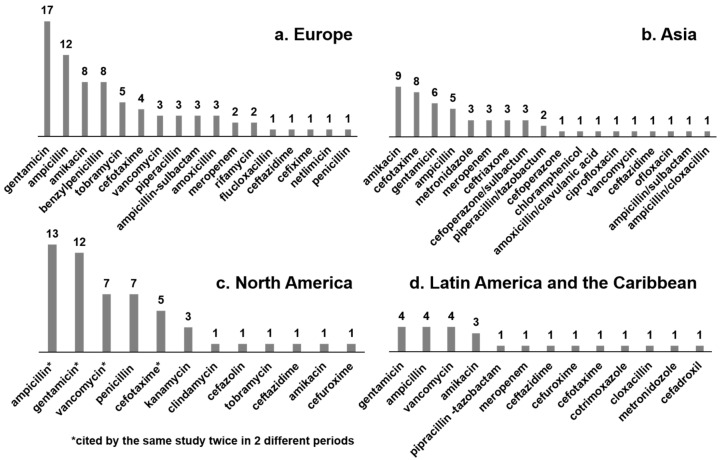
Drugs reported as one of 10 most frequently prescribed antibiotics in neonatal drug utilization studies. (Bars represent the number of studies that reported each drug as one of its 10 most frequently prescribed antibiotic).

Seven studies solely reported the most frequently prescribed antibiotics. In addition, several antibiotics appeared in the list of the most frequently prescribed drugs in studies that did not focus only on antibiotics. In total, 59 studies reported the most frequently used antibiotics. Figure 6 shows the antibiotics and the number of studies that reported it among its most frequently prescribed antibiotic/drug by geographical region. In addition to the data in Figure 6, two studies from Israel [11,45] reported gentamicin, ampicillin and amoxicillin as the most frequently prescribed antibiotics, and one of these [45] also included meropenem among the most frequently prescribed. The two Australasian studies [46,71] included gentamicin, vancomycin, ampicillin and benzylpenicillin in both lists. One African study [6] reported gentamicin, amoxicillin and ceftriaxone as the top three most frequently prescribed antibiotics. The single study from China [55] reported use of cefoperazone-sulbactum, and piperacillin-tazobactum as the most frequently used for all gestational age groups.

## 4. Discussion

This review presents a comprehensive global perspective of neonatal drug utilization research. Over 15 million infants are “born too soon” every year and provision of essential newborn care is imperative for meeting the United Nations’ target to reduce neonatal mortality rates, a key component of the Sustainable Development Goals. Pharmacotherapy plays a large role in neonatal care, particularly intensive care. This role is complicated by several factors including the developmental immaturity of newborn infants, paucity of evidence-base for efficacy, dosing and adverse effects information and the lack of licensed formulations. It is therefore not unsurprising that there is an explosion of interest in this area as reported by Allegaert et al., who found an increasing number of studies investigating drug utilization in newborns [3]. We found drug utilization studies from most parts of the world. Some regions are however sparsely represented—we found only one study from China and one from Africa, both published in the year 2020. India, which has the largest number of preterm births, contributed the largest number of studies, closely followed by the USA. The heightened interest in this area in India is interesting in view of the WHO-led concern that the WHO South-East Asia Region, which includes India, is likely the most at-risk part of the world for the emergence of resistance to microorganisms [80]. Although an increasing number of studies from Europe, and from South America and Australasia, also add to the volume of publications suggesting a world-wide interest, there remains a distinct lack of any collaborative international effort to explore the problem.

Methodologically, the studies remain limited to assessing the most common prescribed drugs either in general, or those that are off-label or unlicensed. Details required to assess the rational use of medications such as indication, dose or duration of use are lacking. Most studies were restricted to single centers and included a limited sample size. Larger studies such as those from the Peadiatrix Medical group in the USA [69,70] are powered by electronic patient records. It is plausible that the use of electronic patient records may enable further large-scale evaluations of drug utilization. This requires efforts to improve electronic patient records such as use of standardized nomenclature and categorization of drugs, collection of data on indications, dosage, adverse effects and medication errors which empower unraveling the yarn of rational prescribing (or the lack of rational prescribing) in neonatal medicine.

The populations included in the studies within this review are quite heterogeneous. Most studies include all neonatal unit admissions with a varied proportion of premature infants. However, it is likely that the composition of the premature cohort is not uniform as studies from high-income countries are likely to include a much more immature population compared to the preterm cohorts in the more resource-limited settings. We found wide variation in the number of medications used per infant ranging from 1.7 drugs per infants reported by Bonati et al. [75] to 11.1 per patient as reported by Neubert et al. [43]. However, we did not see a relationship between the proportion of premature infants included in the study with the average number of drugs prescribed per patient. This is likely to be because of the heterogeneity in the populations and variations in which drugs were excluded from the study. The burden of medication exposure in newborn infants was also well demonstrated by the maximum number of drugs per patient reported in some studies—62 in the most extreme example [38] with several others reporting use of more than 30 drugs in some infants.

We found that the drug utilization pattern is similar across most regions and nations, with a predominance of antibiotics use in all reports. Few studies reported drugs other than an antibiotic as the one in most common usage e.g., caffeine featured at the top of the list in 2 studies. This could be because of the high proportion of premature infants in the study, however we could only confirm this is one study [71] where 87% of included infants were born preterm. Variations in which drugs were excluded from analysis in each study accounts for some other drugs which were not antibiotics appearing as the most frequently prescribed, such as parenteral nutrition, vitamin K and multivitamins which, due to their ubiquitous use, were excluded from most studies. We saw some regional variations: in studies from Asia, specifically India, phenobarbitone was frequently reported. This may reflect the high prevalence of birth asphyxia which, along with prematurity and infections, is one of the three causes reported to account for 0.79 million of 1.01 million neonatal deaths in India in the Million Death Study [81].

The results of this review clearly demonstrate that antibiotics remain the most frequently used drug in neonatal medicine. This is not unexpected as the burden of infections remains high; neonatal sepsis or meningitis accounted for 16% neonatal deaths globally in 2015 [82]. High risk of death and poor outcomes in survivors warrants the reliance on empirical antibiotic usage based on the sensitive but nonspecific clinical diagnosis of possible infections, particularly in preterm infants, and the antibiotics given to clinically well infants born with risk-factors for early-onset sepsis. Unfortunately, the selective pressure exerted by this widespread use is driving antimicrobial antibiotic resistance. Although this is a global problem it is unequally spread, with data from high-income countries such as the UK showing that 95% of pathogens were susceptible to the most commonly used empirical antibiotic regimens, while in low-and middle-income countries up to 70% of pathogens isolated in neonatal sepsis may not be susceptible to the recommended first-line regimens [83]. Many neonates in hospitals in south Asia are now treated with carbapenems as a first-line therapy for sepsis or presumed sepsis [84]. This is reflected in our findings with the more frequent appearance of antibiotics such as third generation cephalosporins and meropenem, and tazobactum in studies from Asia and Latin America. Data from South Asia reflect a high burden on neonatal sepsis and a distinct pathogen profile with predominance of Gram-negative organisms and lower prevalence of group B streptococci as compared to high income countries [85]. In a review of neonatal sepsis in South Asia, Chaurasia et al. reported that 50–88% of common isolates from health facilities are resistant to first-line antibiotics ampicillin and gentamicin and often to third-generation cephalosporins such as cefotaxime. However, most remain susceptible to meropenem and vancomycin, antibiotics that are on the WHO-specified “watch group” [85]. The choice of antibiotics in China as reported by Yue et al. [55] is also unusual when compared to most other countries. Authors suggest that this is driven by the high levels of ampicillin resistance and prohibition of gentamicin use due to the high risk of hearing loss in the population. Against this backdrop, the widespread availability and antimicrobial use in neonates and the contribution of antimicrobial resistance as a complicating factor in neonatal sepsis becomes extremely important and rather than increasing use of antibiotics, infection prevention measures such as hand hygiene, surveillance cultures, contact precautions and antibiotic stewardship should be implemented [86].

Our findings are in keeping with previous reviews. Allegart et al.(2019) [3] which updated the review by Rosli et al. 2017 [87] focused on research objectives, methodology and patterns of drug use across neonatal units. This review also highlighted that antimicrobials such as penicillins and aminoglycosides are amongst the most frequently prescribed drugs to hospitalized infants which is consistent with our findings. Krzyzaniak et al. (2016) also highlighted the frequent report of antibiotics in their included studies [88]. They concluded that patterns of drug utilization were similar across the globe. Our findings, although broadly consistent with this, do demonstrate some variations which may be explained by the difference in disease burden and pattern of antibiotic use in different regions of the world. This difference may be explained by the limited number of studies included in Krzyzaniak et al. In addition, although individually several studies do report this plausible relationship [18,23,32,34,38,43], we did not see a consistent relationship between the proportion of premature infants included in the studies with the number of drugs prescribed per infant as reported by Krzyzaniak et al.. This variation may be because the relationship between prematurity and drug utilization is not straightforward. Moderate to late preterm infants are often well with minimal medical needs while some term infants suffer significant morbidities requiring multiple drugs and prolonged intensive care. The large proportion of term infants who do not require any medications are not admitted to neonatal units and hence are not included in studies where the population is restricted to those in the neonatal unit. In this population, the number of drugs per infant may be more affected by the criteria for admission, range of gestational ages admitted and morbidities in those infants.

Although the included studies have all reported use of medicines prescribed to infants admitted to neonatal units, the studies do not report the admission criteria for their units. Variations such as those in types of neonatal units (for example those providing high levels of intensive care or surgical units vs. special care nurseries) and difference in survival of extremely preterm infants (who form a large part of the work in high-income countries but may not survive beyond a few hours in low-income settings) could account for variations that make any cohesive analysis difficult. The analysis of data extracted from the included studies is limited by the heterogeneity of the included populations, variations in study designs and different methods of reporting the findings. In addition, our review is limited by exclusion of non-English-language studies which may be the reason for missing data or very few reports from some parts of the world.

## 5. Conclusions

We found that the pattern of drug utilization in neonatal units is largely similar across global regions. A few exceptions reflect the patient population included in the study, differences in the burden of neonatal pathologies and the variations in antibiotic usage reflect the global burden of antimicrobial resistance. The review also highlights the lack of details such as paucity on information indication, dose and duration of use or adverse effects, calling for improved collection and analysis of drug utilization data in neonatal medicine. Such research, particularly when conducted collaboratively across national and continental boundaries, is imperative to promote rational use of medicine in neonates.

## 6. Patents

Not Applicable.

## Figures and Tables

**Table 1:** Drugs reported to be among the 10 most frequently prescribed in neonatal drug utilization studies in different regions across the world.

Geographic Region (Number of Studies) (Ref)	Most Frequently Prescribed Drugs (Number of Studies Citing the Drug among the 10 Most Frequently Prescribed Drugs)
**Europe (24 studies)** [7,13,20,25,26,29,31,35,37,42,43,44,50,59,62,63,65,67,68,71,74,75,76,79]	caffeine (18 studies), gentamicin (17 studies), ampicillin (11 studies), furosemide (9 studies), multivitamins (9 studies), vitamin K (11 studies), benzylpenicillin (8 studies), amikacin (6 studies), morphine (5 studies), paracetamol (6 studies)
**North America (10 studies)** [8,9,28,38,53,60,69,70,72,78]	ampicillin (8 studies), gentamicin (8 studies), furosemide (6 studies), surfactant (6 studies), penicillin (5 studies), vancomycin (6 studies), caffeine citrate* (6 studies), cefotaxime (4 studies), dopamine (5 studies), calcium gluconate (4 studies)
**Asia (6 studies)** [14,17,19,51,55,73]	phenobarbitone (4 studies), vitamin K (4 studies), amikacin (3 studies), aminophylline (3 studies), ceftriaxone (2 studies), ceftazidime (2 studies), gentamicin (2 studies), phenytoin (2 studies), penicillin/sulbactam (2 studies),caffeine (1 study)
**Latin America and Caribbean (4 studies)** [15,24,32,41]	fentanyl (4 studies), gentamicin (3 studies), vancomycin (3 studies), multivitamins (3 studies), amikacin (2 studies), ampicillin (2 studies), furosemide (2 studies), aminophylline (2 studies), morphine (1 study), metamizole (1 study)
**Middle East (2 studies)** [11,45]	gentamicin, ampicillin, amoxicillin, vitamins
**Australasia (2 studies)** [22,46]	vancomycin, gentamicin

## Appendix A. Search Strategy

**Table A1:** Search strategy for drug utilization review (from inception of database to February 2019 using OR, AND).

Database	Search Terms	Combination of Search Terms (A Combination between Title Abstract Free Text Keywords and Mesh Terms)	Number of Hits
**EMBASE** **(provided by Ovid)** **From 1974 to 9 April 2019**	**Population search terms:** Free text words: Infant*-newborn*-neonate* MeSH terms: INFANT-NEWBORN **Drug utilization search terms:** Free text words: “drug use”-drug utilization MeSH terms: DRUG UTILIZATION-”DRUG USE” **Setting search terms:** Free text words: neonatal intensive care unit*-neonatal unit* MeSH terms: NEWBORN INTENSIVE CARE- NEONATAL INTENSIVE CARE UNIT	~”(((infant*).ti,ab OR (newborn*).ti,ab OR (neonate*).ti,ab OR *INFANT/OR exp INFANT/OR *NEWBORN/) AND ((“drug use”).ti,ab OR (“drug utilization”).ti,ab OR *”DRUG UTILIZATION”/OR exp “DRUG UTILIZATION”/OR *”DRUG USE”/OR exp “DRUG USE”/)) AND (*”NEWBORN INTENSIVE CARE”/OR *”NEONATAL INTENSIVE CARE UNIT”/)”	232
**Medline** **(provided by ProQuest)** **From 1946 to 9 April 2019**	**Population search terms:** Free text words: Infant*-newborn*-neonate* MeSH terms: INFANT-INFANT, NEWBORN **Drug utilization search terms:** Free text words: “drug use”-drug utilization MeSH terms: DRUG UTILIZATION **Setting search terms:** Free text words: neonatal intensive care unit*-neonatal unit* MeSH terms: Care,neonatal intensive- intensive care units,neonatal infant,newborn,intensive care-neonatal intensive care-neonatal intensive care units	~”(((infant*).ti,ab OR (neonate*).ti,ab OR (newborn*).ti,ab OR *INFANT/OR exp INFANT/OR *”INFANT, NEWBORN”/OR exp “INFANT, NEWBORN”/) AND ((“drug use”).ti,ab OR (drug utilization).ti,ab OR *”DRUG UTILIZATION”/OR exp “DRUG UTILIZATION”/OR *”DRUG UTILIZATION REVIEW”/OR exp “DRUG UTILIZATION REVIEW”/)) AND ((neonatal intensive care unit*).ti,ab OR (neonatal unit*).ti,ab OR *”INTENSIVE CARE UNITS, NEONATAL”/OR exp “INTENSIVE CARE UNITS, NEONATAL”/)”	254
**CINAHL** **(provided by EBSCO)** **From 1937 to 9 April 2019**	**Population search terms:** Free text words: Infant*-newborn*-neonate*MeSH terms: INFANT-INFANT,NEWBORN **Drug utilization search terms:** Free text words: “drug useinfa”- drug utili?ationMeSH terms:DRUG UTILIZATION **Setting search terms:** Free text words: neonatal intensive care unit*-neonatal unit*MeSH terms: INTENSIVE CARE UNITS,NEONATAL	Combination of search terms(A combination between title abstract key words, and Mesh terms was done for a compressive search from inception of the database to February 2019 using OR, AND) ~”(((infant*).ti,ab OR (newborn*).ti,ab OR (neonate*).ti,ab OR *INFANT/OR exp INFANT/OR *”INFANT, NEWBORN”/OR exp “INFANT, NEWBORN”/) AND ((“drug use”).ti,ab OR (drug utilization).ti,ab OR *”DRUG UTILIZATION”/)) AND ((neonatal unit*).ti,ab OR (neonatal intensive care unit*).ti,ab OR *”INTENSIVE CARE UNITS, NEONATAL”/OR exp “INTENSIVE CARE UNITS, NEONATAL”/)”	87
**Total hits**	573		

## Appendix B. Characteristics of Included Studies

### Appendix B.1. Description of Drug Utilization Studies on Drug Use in All Categories (60 Studies)

**Table A2:** Studies of drug utilization in Europe (27 studies).

Study ID	Study Period	Inclusion and Exclusion Criteria	Number of Neonates (% Female)	Gestation Age (Weeks) Birth Weight (Grams)	Number of Drugs Per Neonate
**Italy (6 studies)**					
**Bonati** **1988 (75)**	One year (year not reported)	Inclusion: all admitted neonates Exclusion: fluids and electrolytes, glucose, oxygen, vitamin K and prophylactic ophthalmic preparation	n = 706 (47%)	GA (mean, range): 33.3, 26–36 BW (mean, range): 2013, 510–3600	Mean (SD): 1.7 (0–8)
**Dell’ Aera 2007 (25)**	July–August 2004	Inclusion: all admitted neonates Exclusion: not reported	n = 34 (not reported)	Not reported	Not reported
**Dessi** **2010 (26)**	March 2007 (1 month)	Inclusion: all admitted neonates receiving drugs Exclusion: saline, blood transfusions, oxygen	n = 38 (not reported)	GA not reported BW not reported	Range: 1–4
**Laforgia** **2014 (67)**	May 2011 (1 month)	Inclusion: all admitted neonates with at least one drug Exclusion: not reported	n = 126 (not reported)	GA (median, range): 31, 23–36 BW not reported	Median (range): 3 (1–7)
**Cuzzolin** **2016 (71)**	May–July 2014	Inclusion: all admitted neonates with at least one drug Exclusion: not reported	n = 220 (41%)	Not reported	Median (range): 4 (1–9)
**Girardi** **2017 (31)**	January 2009–December 2011 (3 years)	Inclusion: all admitted neonates with GA < 37 weeks and BW ≤ 1500 g Exclusion: died within the first 48 h after birth	n = 159 (not reported)	Mean (range) 1000–1500 g group: 30 (27–36) <1000 g group: 26 (22–33)	Not reported
**Spain (4 studies)**					
**Martinez** **2005 (42)**	October–December 2003	Inclusion: all admitted neonates Exclusion: not reported	n = 48 (not reported)	Not reported	Mean (range): 3.9 (1–14)
**Payares** **2010 (47)**	8 months (year not reported)	Not reported	n = 52 (48%)	GA: 0–48 days BW (range): 550–3920	Not reported
**Blanco-Reina** **2016 (13)**	July–November (year not reported)	Inclusion: admitted neonates with at least one drug Exclusion: not reported	n = 48 (41%)	GA (mean (SD)): 34.5 (4.2) BW (mean (SD)): 2335 (949)	Mean (SD): 7.4 (6)
**Alonso** **2019 (7)**	April–September 2018	Inclusion: all admitted neonates Exclusion: blood products, TPN, fluids and oxygen	n = 84 (38%)	Not reported	Not reported
**France (4 studies)**					
**Gouyon 2019** **(74)**	January 2017–December 2018	Inclusion: all patients with a first prescription before 28th day of life and at least one electronic medication prescription Exclusion: no prescriptions, or none in first 28 days; handwritten prescriptions only	n = 27382 (55%)	GA (mean (SD)): 35.4 (4.3) BW (mean (SD)): 2457.8 (944.5)	Mean (SD): 6.2 (5.7)
**Gortner** **1991 (77)**	August 1989–May 1990 (10 mo)	Inclusion: premature neonates with a need of intubation and mechanical ventilation Exclusion: vitamin K and heparin	n = 164 (46%)	GA (mean (SD)): 27.2 (1.2) BW (mean (SD)): 970 (145)	Not reported
**Nguyen** **2011 (44)**	January–April 2009	Inclusion: all admitted neonates Exclusion: TPN, IV fluids, oxygen and drugs used in research studies	n = 65 (not reported)	GA (median(range)): 34 (27–41) BW median(range)) 1930 (810–4520)	Median (range): 4 (1–7)
**Riou** **2015 (62)**	1 year (2012)	Inclusion: all admitted neonates with at least one drug Exclusion: blood products, oxygen therapy, enteral and parenteral nutrition, and standard intravenous replacement solutions	n = 910 (43%)	GA (median (IQR)): 34 (31–37) BW (median (IQR)): 2040 (1530–2270)	Median (IQR): 8 (5–13)
**The Netherlands (3 studies)**					
**Jong** **2001 (35)**	February–March 1999	Inclusion: all admitted neonates Exclusion: blood products, TPN, oxygen therapy, IV fluids	n = 64 (50%)	Not reported	Not reported
**Flint** **2014 (29)** **(abstract)**	January 2007–June 2013	Not reported	n = 4054 (45%)	GA (median, range): 32, 23^+6^–42^+2^ BW median, range): 1800, 360–5400)	Not reported
**Flint** **2018 (76)**	September 2014–August 2015 (1 year)	Inclusion: all admitted neonates Exclusion: electrolytes, TPN, vaccines, dermatological products, contrast media	n = 1491 (48%)	GA (median, IQR): 32^+5^, 29^+6^ to 37^+6^ BW (median (IQR)): 1865,1253–3000	Median (IQR):5 (3–10)
**Germany (2 studies)**					
**Lindner** **2008 (61)**	Study period not reported)	Inclusion: all neonates with GA < 32 Exclusion: not reported	n = 113 (44%)	GA (mean (SD)): 26.9 (1.65) BW (mean (SD)) 930 (253)	Not reported
**Neubert** **2009 (43)**	December 2004–October 2005 (11 months)	Inclusion: all admitted neonates for >24 h Exclusion: IV infusions (e.g., glucose or chloride), TPN and oxygen	n = 183 (44%)	GA (mean (SD)): 33.6 (4.66) BW (mean (SD)): 2134 (935)	Mean (SD): 11.1 (9.56) Range: 0–45
**UK (2 studies)**					
**Conroy** **1999 (20)**	February–May 1998 (13 weeks)	Inclusion: all admitted neonates Exclusion: IV fluids, flushes of sodium chloride 0.9% or heparin, blood products (other than albumin) and oxygen therapy	n = 70 (not reported)	GA (preterm only) (median, range): 33 (26 to 36)	Median (range): 3.5 (0–42)
**Turner** **2009 (79)**	December 2007–April 2008	Inclusion: all admitted neonates Exclusion: blood products, IV fluids, TPN	Not reported	Not reported	Not reported
**Ireland (one study)**					
**Kieran** **2013 (37)**	February–March 2012	Inclusion: all admitted neonatesExclusion: not reported	n = 110 (not reported)	GA (mean (SD)): 35 (5) BW (median, (IQR)): 2615 (1601 -3500)	Median (IQR): 4 (3–11)
**Portugal (one study)**					
**Silva** **2015 (50)**	January–June 2013	Inclusion: all admitted neonates Exclusion: oxygen, IV fluids and flushes, drugs used in surgeries, enteral and parenteral nutrition, contrast agents, vaccines, blood products (except albumin and immunoglobulins), basic creams, drugs on clinical trials	n = 218 (45%)	GA (mean (SD)): 36.07 (4.0) BW (mean (SD)): 2554 (910.5)	Median (range): 3 (0–34)
**Estonia (one study)**					
**Lass** **2011 (59)**	February–August 2008	Inclusion: all admitted neonates Exclusion: IV fluids, blood products, oxygen, nutritional and technical products, basic creams and ointments, TPN, vaccines and vitamins	n = 490 (not reported)	GA not reported BW (mean (SD)): 2446 (1124)	Median (IQR, max): 4 (2–7, 27)
**Slovak Republic (one study)**					
**Schweigertova** **2016 (63)**	April–September 2012	Inclusion: all admitted neonates Exclusion: IV replacement solutions, TPN, vaccines, blood products and oxygen	n = 202 (49%)	GA (mean (SD)): 36 (3.4) BW: Not reported	Mean (SD): 4.8 (2.7)
**Turkey (one study)**					
**Oguz** **2012 (68)**	December 2011	Inclusion: all admitted neonates Exclusion: standard IV solutions, sodium chloride 0.9% infusions, TPN, blood products (except albumin) and oxygen	n = 93 (not reported)	GA (mean (SD)): 32.5 (4.7) BW (mean (SD)): 2081 (951)	Median (range): 3 (1–11)
**Multi-European countries (21 countries)**					
**Mesek** **2019 (65)**	January–June 2012	Inclusion: all infants in the neonatal unit and receiving prescription on the day (at 8 AM) Exclusion: blood products, glucose and electrolyte solutions, vaccines, nursery care topical agents, herbal medicines and enteral nutrition including breast milk fortifiers	n = 726 (43%)	GA (median, (IQR)): 34 (30–38) BW (median, (IQR)): 1993 (1356–3006)	Not reported
**BW, birthweight; GA, gestation age; IV, Intravenous; SD, standard deviation; TPN, total parenteral nutrition**					

**Table A3:** Studies of drug utilization in Middle East (2 studies).

Study ID	Study Period	Inclusion and Exclusion Criteria	Number of Neonates (% Female)	Gestation Age (Weeks) Birth Weight (Grams)	Number of Drugs Per Neonate
**Israel (2 studies)**					
**Barr** **2002 (11)**	April–July 2000	Inclusion: all admitted neonates Exclusion: saline, heparin flush, blood transfusions, and oxygen	n = 105 (not reported)	Not reported	Range: 1–13
**Nir-Neuman** **2018 (45)**	December 2015–January 2016	Inclusion: all admitted neonates Exclusion: TPN, blood products, fluids, oxygen therapy, nasal sprays, eye drops, ointments, and local creams	n = 134 (49%)	GA (median, IQR): (35, 33–38) BW (mean(SD)): 2424 (854)	Median (IQR): 6 (2–17)
**BW, birthweight; GA, gestation age; SD, standard deviation; TPN, total parenteral nutrition**					

**Table A4:** Studies of drug utilization in North America (12 studies).

Study ID	Study Period	Inclusion and Exclusion Criteria	Number of Neonates (% Female)	Gestation Age (Weeks) Birth Weight (Grams)	Number of Drugs Per Neonate
**USA (9 studies)**					
**Russel** **1983 (79)**	Not reported	Not reported	Not reported	Not reported	Not reported
**Lesko** **1990 (60)**	1978–1986	Inclusion: all admitted for >24 h Exclusion: vitamin K and topical products such as silver nitrate	n = 2690 (43%)	GA: not reported BW (median): 2220	8
**Clark** **2006 (70)**	February–May 1998 (13 weeks)	Inclusion: all neonates in database Exclusion: Not reported	n = 253651 (44%)	GA (median, IQR): 35 (33–38) BW (median, IQR): 2460 (1790–3200)	Not reported
**Du** **2006 (28)**	January 1997–June 2004 (7 years) 2 periods 1st: 1997–1998 2nd: 2001–2004	Inclusion: all admitted neonates with at least one drug Exclusion: TPN, oxygen administration, vitamin K prophylaxis, erythromycin ophthalmic prophylaxis, routine cord care, vaccinations, blood and blood products (except fresh frozen plasma)	1st period: n = 2332 (47%) 2nd period: n = 2691 (44%)	1st period:GA (mean): 35.7 BW (mean): 2580 2nd period: GA (mean): 35.3 BW (mean): 2499	Median (range) 1st period: 3.37 (2, 1–28) 2nd period 3.72 (2, 1–36)
**Warrier** **2006 (53)**	January 1997–June 2004	Inclusion: all admitted neonates with at least one drug Exclusion: blood and blood products (except fresh frozen plasma), TPN, oxygen, vitamin K prophylaxis, erythromycin ophthalmic prophylaxis, routine cord care, vaccinations, TPN, normal saline except when treatment for hypotension	n = 6839 (46%)	GA (mean (SD)): 35 (5) BW (mean (SD)): 2498 (1000)	Mean (SD): 3.6 (3.9)
**Kumar** **2008 (38)**	September 2000–August 2003 (3 years)	Inclusion: all admitted neonates Exclusion: TPN, nutritional supplements such as vitamins, standard intravenous fluids, immunizations and drugs used in research studies	n = 2304 (43%)	GA (mean (SD)): 34.1 (4.6) BW (mean (SD)): 2325 (1014)	Mean (SD): 8.5 (8.3)
**Hsieh** **2014 (69)**	2005–2010 (5 years)	Inclusion: all admitted neonates Exclusion: after a day of life 120, and all vitamins (except vitamin A), nutritional supplements, vaccines, eye drops and topical medications.	n = 450,386 (44%)	GA (median, IQR): 35 (33–38) BW (median, IQR) 2490 (1830 to 3191)	Mean (range): 4 (1–14) For <1000 gm Mean (range): 17 (2–45)
**Gulati** **2016 (33)**	1990–2011 (22 years)	Inclusion: all very low BW neonates Exclusion: volume boluses, blood and blood products, TPN, and topical medications	n = 5529 (50%)	GA (median, IQR): 28 (26–30) BW (median, IQR): 1017 (745–1271)	Median (IQR): 9 (5–15)
**Puia-Dumitrescu** **2020 (72)**	2006–2016 (10 years)	Inclusion: 22–24 week admitted to the NICU without major congenital anomalies Exclusion: missing or incomplete discharge data or discharge home at <32 weeks postnatal age. All nutritional supplements, vitamins (except Vitamin A), vaccines, eye drops and topical medications	n = 7578 (47%)	GA: Not reported BW (median, (IQR)): 610 (540–680)	Median (IQR): 13 (8, 18)
**Canada (3 studies)**					
**Aranda** **1982 (8)**	Not reported	Inclusion: all admitted neonates Exclusion: Drugs for routine prophylaxis (e.g., antimicrobial eye drops)	n = 293 (not reported)	GA (mean (SD)): 36.4 (0.25) BW (mean (SD)): 2687 (157)	Mean (SD): 6.2 (5.7) Range: 1–26
**Aranda** **1983 (9)**	2 periods; 1st: July 1974–February 1975 2nd: February 1977–November 1977	Inclusion: all admitted neonates Exclusion: vitamin K, ophthalmic preparations, fluids and electrolytes, IV amino acids/intralipids and/or glucose (except if for neonatal hypoglycaemia, phototherapy and oxygen	Not reported	GA (mean (SD)): 1st period: 36.9 (0.2); 2nd period: 36.42 (0.25); BW (mean (SD)): 1st period: 2612 (51) 2nd period: 2686.9 (156.7)	Mean (SD): 1st period: 3.40 (0.20) 2nd period: 6.19 (0.33)
**Collinge** **1988 (21)**	Not reported	Inclusion: all admitted neonates Exclusion: not reported	n = 1200 (not reported)	Not reported	5.7
**BW, birthweight; GA, gestation age; SD, standard deviation; TPN, total parenteral nutrition**					

**Table A5:** Studies of drug utilization in Asia (11 studies).

**Study ID**	**Study Period**	**Inclusion and Exclusion Criteria**	**Number of Neonates** **(% Female)**	**Gestation Age (Weeks)** **Birth Weight (Grams)**	**Number of Drugs Per Neonate**
**China (one study)**					
**Yue 2020** **(55)**	March–Apr 2018	Inclusion: all inpatients Exclusion: IV solutions (including 0.9% sodium chloride, 5% or 10% glucose injection, and sterile solution for injection), blood products (except albumin), 1% silver nitrate eye drops, parenteral nutrition,, heparin for venous access, oxygen, and electrolytes (such as calcium gluconate, sodium bicarbonate, magnesium sulphate and potassium chloride)	n = 319 (44%)	GA (mean (SD)): 35.8 (3.9) BW (mean (SD)): 2570 (911)	Median (IQR): 3 (1, 5.5)
**India (9 studies)**					
**Chatterjee** **2007 (17)**	Inclusion: all admitted neonates Exclusion: not reported	n = 176 (37%)	GA: not reported BW (mean (SD)): 2214 (774)	4.8
**Sharanappa** **2014 (48)**	January–June 2013	Not reported	n = 100 (not reported)	Not reported	Not reported
**Brijal** **2015 (14)**	March 2013–February 2014 (1 year)	Inclusion: all admitted neonates Exclusion: neonates who are discharged or die within 24 hours of NICU admission	n = 650 (38%)	GA: not reported BW (mean (SD)): 2160 (600)	4.46
**Suryawanshi** **2016 (51)**	April–September 2014	Not reported	n = 528 (39%)	GA (mean (SD)): 35 (3) BW (mean (SD)): 2000 (700)	Mean (SD): 4.37 (2.91)
**Chauthankar** **2017 (18)**	July 2014–March 2015 (9 mo)	Inclusion: all admitted neonates with at least one drug Exclusion: blood, blood products, vitamin K prophylaxis, prophylactic ophthalmic treatment, vaccines or IV fluids	n = 460 (41%)	GA not reported BW (mean (SD)): 2000 (700)	Mean (SD) 5.7 (3.6)
**Choure** **2017 (19)**	April–September 2014 (6 mo)	Inclusion: all admitted neonates Exclusion:IV fluids, parenteral nutrition, nutritional supplements, blood and blood products, oxygen, phototherapy and vaccinations	n = 220 (46%)	Not reported	Mean (range): 3.6 (1–6)
**Jayaram** **2017(34)**	August–January 2016 (6 mo)	Inclusion: all admitted neonates Exclusion: IV fluids, TPN, routine oral nutritional supplements, vaccines, Vitamin K, topical anaesthetic cream, oxygen and blood products	n = 154 (46%)	GA (mean(SD)): 34 (2.75) BW (mean(SD)): 1712 (914)	Mean (SD): 8.4 (7.6) Range: 0–17
**Ashwin** **2018 (10)**	6 months (year not reported)	Not reported	n = 70 (39%)	GA (mean (SD)): 35 (3.14) BW mean (SD)) 2200 (730)	Mean: 3
**Kumari** **2019 (39)**	October 2017–Dec 2017	Inclusion: all admitted neonates Exclusion: IV fluids, vaccines, Vitamin K, oxygen, and blood products	n = 81 (33%)	GA: not reported BW (mean): 2261	Mean (range): 6.9 (1–14)
**Pakistan (one study)**					
**Aamir** **2018 (73)**	March–August 2005 (6 mo)	Inclusion: all admitted neonates Exclusion: topical medication, oxygen, IV solution	n = 1300 (32%)	GA (median, range): 33, 26–35 BW: not reported	Mean (SD): 2.85 (1.358) Range: 1–9
**BW, birthweight; GA, gestation age; SD, standard deviation; TPN, total parenteral nutrition**					

**Table A6:** Studies of drug utilization in Latin America and Caribbean (6 studies).

Study ID	Study Period	Inclusion and Exclusion Criteria	Number of Neonates (% Female)	Gestation Age (Weeks) Birth Weight (Grams)	Number of Drugs Per Neonate
**Brazil (5 studies)**					
**Marino** **2011 (41)** **(abstract)**	January 2006–December 2007	Not reported	n = 827 (not reported)	4 groups: a: <1000 g b: 1000–1499 c (1500 to 2499) d: ≥2500 g	Group a: 11.1 Group b: 6 Group c: 1.7 Group d: 1.2
**Carvalho** **2012 (15)**	July–August 2011	Inclusion: all admitted neonates Exclusion: blood and blood products, parenteral nutrition, oxygen and other gases, vit K, silver nitrate, vaccines	n = 61 (41%)	Not reported	5
**Gonçalves** **2015 (32)**	January–June 2012	Inclusion: all admitted neonates for more than 24 h Exclusion: sodium chloride, 5% glucose, blood products (except albumin), heparin vaccines, phytonadione, 1% silver nitrate eye drops, TPN, oxygen, and electrolytes	n = 187 (42%)	GA (median, IQR): 36.6,33.9–38.3 BW (mean(SD)): 2473 (831)	Mean (range): 6.4 (0–40)
**De Souza Jr** **2016 (24)**	6 months (year not reported)	Inclusion: neonates with electronic records of more than 24 h who drug Exclusion: incomplete clinical data, vaccines, blood products, TPN, silver nitrate eye drops or IM vitamin K in delivery room or IV fluids	n = 192 (50%)	GA (mean (SD)): 33.3 (4.3) BW (mean (SD)): 1909.5 (886)	Mean (SD): 8.8 (6.1)
**De Lima Costa** **2018 (50)**	August 2015–July 2016	Inclusion: all admitted neonates Exclusion: TPN, IV fluids, oxygen, blood products or electrolytes	n = 220 (46%)	GA (mean (SD)): 32.4 (4.4) BW (mean (SD)): 1932.7 (1127.6)	Mean (SD): 8.2 (6.2) Range: 1–33
**Argentina (one study)**					
**Fungo** **2013 (30)**	January–December 2011	Inclusion: not reported Exclusion: preparations made locally or donated or acquired by family	Not reported	Not reported	Not reported
**BW, birthweight; GA, gestation age; IV, intravenous; SD, standard deviation; TPN, total parenteral nutrition**					

**Table A7:** Studies of drug utilization in Australasia (2 studies).

Study ID	Study Period	Inclusion and Exclusion Criteria	Number of Neonates (% Female)	Gestation Age (Weeks) Birth Weight (Grams)	Number of Drugs Per Neonate
**New Zealand (one study)**					
**Daniell** **1989 (22)**	November 1987–February 1988	Inclusion: all admitted neonates Exclusion: IV glucose, TPN, oxygen, blood products, sodium chloride flush, expressed milk and milk formula	n = 79 (not reported)	GA (mean (SD)): 34 (0.6) BW (mean (SD)): 2185 (112)	Mean (SD): 8.6 (0.9) Range: 0–30
**Australia (one study)**					
**O’Donnell** **2002 (46)**	December 2001–February 2002	Inclusion: all admitted neonates Exclusion: TPN, IV fluids, oxygen, and drugs used in research studies	n = 97 (not reported)	GA (median, range): 31, 22.7–41.1 BW (median, range): 1560, 414- 4790	Not reported
**BW, birthweight; GA, gestation age; IV, intravenous; SD, standard deviation; TPN, total parenteral nutrition**					

### Appendix B.2. Description of Drug Utilization Studies for Antibiotics Use Only (11 Studies)

**Table A8:** Studies of drug utilization on antibiotics only (11 studies).

Study ID	Study Period	Inclusion and Exclusion Criteria	Number of Neonates (% Female)	Gestation Age (Weeks) Birth Weight (Grams)	Number of Drugs Per Neonate
**Asia -India (4 studies)**					
**Gandra 2018**	February 2016–February 2017 (one year)	Inclusion: all admitted neonates with active antimicrobial prescriptions Exclusion: not reported	n = 403 (32%)	GA (median, IQR): 34.5 (31–38) BW (median (IQR): 1737, 1210–2710	Not reported
**Hauge 2017**	April 2008–March 2010 (3 years)	Inclusion: neonates with sepsis Exclusion: not reported	Hospital: Teaching: 217 (63%) Non-teaching: 1572 (49%)	Not reported	Teaching 7 Non-teaching: 4
**Shinde** **2017 (49)**	October 2011–September 2012	Inclusion: neonates with sepsis Exclusion: discharged or transferred or died within 2 days in NICU	N= 84 (29%)	GA: not reported BW (mean (SD)): 2000(620)	Not reported
**Subash** **2015 (52)**	February–April 2013	Inclusion: suspected or diagnosed sepsis Exclusion: surgical problems, major congenital malformations, on antibiotics or if mother received antibiotics before delivery	n = not reported (42%)	Not reported	Not reported
**Latin America and Caribbean-Trinidad and Tobago (one study )**					
**Hariharan 2013**	September–November 2008	Inclusion: all infants receiving antimicrobials Exclusion: not receiving antimicrobials	n = 353 (not reported)	GA: <40 weeks BW (mean (SD)): 2960 (940)	Not reported
**Middle East- Saudi Arabia (one study)**					
**Balkhy** **2019 (54)**	October 2012–June 2013 (33 mo)	Inclusions: <16 years (data on neonates reported separately) who received at least one antimicrobials Exclusion: antimicrobial by route other than parenteral or oral routes	n = 1813 (not reported)	Not reported	Not reported
**Africa-Zimbabwe (one study)**					
**Chiminhi** **2020 (6)**	May–November 2018	Inclusion: all admitted infants Exclusion: none mentioned	n = 459 (49%)	GA: not reported BW (median, (IQR)): 2800 (2–3.4)	Not reported
**Latin America and Caribbean-Chile (one study)**					
**Jimenez 2017**	Four years	Inclusion: all admitted infants Exclusion: not reported	n = 5,619 (46.5%)	GA (mean (SD)): 36.2 (3.6) BW: not reported	Not reported
**North America-USA (2 studies)**					
**Cantey** **2015 (5)**	October 2011–November 2012 (4 mo)	Inclusion: all neonates admitted to NICU Exclusion: not reported	Retrospective: 593 (57%) Prospective: 1014 (43%)	GA (median, IQR): Retrospective: 38, 34.5–39.4 Prospective: 37.4, 34.1–39.1 BW (median, IQR) Retrospective: 2860, 2145–3457 BW (median, IQR): 2793, 2070–3435	Not reported
**Grohskopf 2005**	August 1999–February 2000	Inclusion: infants admitted at NICU at each participating hospital on study dates Exclusion: not reported	n = 1580 (45%)	Not reported	Median (range) 2 (1–5)
**Europe-Brazil and Germany (one study)**					
**Silva** **2020 (50)**	January–December 2018 (Brazil) May–August 2016 (Germany)	Inclusion: neonatal or paediatric intensive care unit admissions, had antimicrobial for >24h. Exclusion: topical and inhaled antibiotics	n = 2567 (not reported)	Not reported	Not reported
**BW, birthweight; GA, gestation age; NICU; neonatal intensive care unit; PPS, point prevalence survey; SD; standard deviation**					

### Appendix B.3. Description of Drug Utilization Studies on Off-Label and Unlicensed Drugs Only (6 Studies)

**Table A9:** Studies of drug utilization on off-label and/unlicensed drugs only (6 studies).

Study ID	Study Period	Inclusion and Exclusion Criteria	Number of Neonates (% Female)	Gestation Age (Weeks) Birth Weight (Grams)	Number of Drugs Per Neonate
**Europe- Spain (one study)**					
**Casan 2017**	November 2015–February 2016	Inclusion: all admissions Exclusion: crystalloid fluids, plasma-expanding serums (except for albumin), parenteral nutrition, antiseptics, and heparins for preventing catheter block	n = 41 (32%)	GA (mean (SD)): 35.9 (4.22) BW (mean (SD)): 3280 (860)	Mean (SD): 6.65 (3.28)
**North America-Canada (one study)**					
**Doherty** **2010 (27)**	May 2009 (1 mo)	Inclusion: all admissions Exclusion: not reported	n = 38 (53%)	Not reported	Not reported
**Asia-India (one study)**					
**Jain** **2014 (57)**	June–August 2009	Inclusion: all inpatients for >6 h and had a drug Exclusion: nutritional supplements, IV fluids, inotropes, vaccines, vitamin K, topical anaesthetic cream, fluid or heparin for flushing IV lines, oxygen and blood products	n = 156 (not reported)	GA (median, IQR): 32, 30–35 BW (median, IQR): 1348, 1076–1800	Median (IQR): 6, 1–6
**Middle East -Iran (one study)**					
**Kouti** **2019 (58)**	January–March 2016 (3 mo)	Inclusion: admitted for at least 24 h who received at least one medication Exclusion: oxygen, vaccines, blood products (except immunoglobulin), vitamins, electrolytes, TPN, IV fluids	n = 193 (41%)	GA (mean (SD)): 34 (4.4) BW (mean (SD)): 2463 (955)	Mean (SD): 4.5 (3) Range: 1–17
**Middle East-Saudi Arabia (one study)**					
**Mazhar** **2018 (40)**	January–March 2015 (3 mo)	Inclusion: all admitted neonates for a minimum of 24 h and prescribed at least one drug	n = 138 (48%)	GA (median, IQR): 35, 35–39 BW: not reported	Mean (SD): 3.5 (2.3)
**Africa-Ethiopia (one study)**					
**Gidey** **2020 (56)**	March–April 2019	Inclusion: admitted for at least 24h; prescribed at least one medication Exclusion: oxygen therapy, parenteral nutrition, blood products, antiseptics, vaccines and IV fluid (e.g., normal saline, dextrose); incomplete information in their prescription	n = 122 (41%)	GA: not reported BW (mean (SD)): 2540 (790)	Mean (SD): 3.02 (1.40)
**BW, birthweight; GA, gestation age; IV; intravenous; NICU; neonatal intensive care unit; SD; standard deviation**					

### Appendix B.4. Description of Drug Utilization Studies on Specific Pharmacologic Groups Only (7 Studies):

**Table A10:** Studies of drug utilization on specific pharmacologic groups only (5 studies).

Study ID	Study Period	Inclusion and Exclusion Criteria	Number of Neonates (% Female)	Gestation Age (Weeks) Birth Weight (Grams)	Number of Drugs Per Neonate
**North America-USA (one study)-Antiepileptics**					
**Ahmad** **2017 (64)**	January 2005–December 2014	Inclusion: all with diagnosis of seizure or seizure disorder and received one of the following AED: phenobarbital, phenytoin/fosphynytoin/levetiracetam, topiramate, lidocaine or carbamazepine Exclusion: benzodiazepine for sedation	n = 9134 (42%)	GA (mean (SD)): 34.8 (5.8) BW (mean (SD)): 2500(1200)	Not reported
**North America-Canada (one study)-Sedatives and narcotics**					
**Toye 2018**	2004–2009	Inclusion: born at <35 weeks of GA admitted to NICUs contributing data to Canadian Neonatal Network 2004–2009 Exclusion: not reported	n = 12,415 (not reported)	Not reported	Not reported
**North America-USA (one study)-Drugs used in BPD**					
**Bamat 2019**	January 2007–August 2016	Inclusion: diagnosed with symptomatic bronchopulmonary dysplasia Exclusion: born at ≥32 weeks of GA, admitted after 36 weeks postmenstrual age; admitted for <1 week	n = 3252 (40%)	GA (median, (IQR)): 26 (24–28) BW (median, (IQR)): 790 (640–1040)	Range: 22–50
**Europe-Spain (one study)-Sedatives and analgesics**					
**Avila-Alvarez** **2015 (7)**	November 2012 (one mo)	Inclusion: all admissions during study period with corrected age of 44 Exclusion: not reported	n = 468 (45%)	GA (mean (SD)): 34.3 (4.6) BW (mean (SD)): 2182 (9764)	Not reported
**Europe-France (one study)-Analgesics**					
**Benahmed-Canat** **2019 (12)**	January 2012–June 2013	Inclusion: all infants undergoing surgery Exclusion: not reported	n = 168 (40%)	GA (mean (SD)): 35.1 (4.6) BW (mean (SD)): 2337(1006)	Mean (SD): 2.6 (1.3)
**Europe-Estonia (one study)-Cardiovascular drugs**					
**Hallik 2014** **(abstract)**	Not reported	Not reported	n = 726 (not reported)	GA (median, range): 34, 23–42 BW (median, range): 1993, 400–4720	Not reported
**Europe-Spain (one study)-Intravenous drugs**					
**De Basagoiti** **2019 (84)**	January–February 2018	Not reported	Not reported	Not reported	Not reported
**BW, birthweight; GA, gestation age; NICU; neonatal intensive care unit; IQR, interquartile range; SD; standard deviation**					
